# Methylomic profiling in trisomy 21 identifies cognition- and Alzheimer’s disease-related dysregulation

**DOI:** 10.1186/s13148-019-0787-x

**Published:** 2019-12-16

**Authors:** Larissa Haertle, Tobias Müller, Roy Lardenoije, Anna Maierhofer, Marcus Dittrich, Renzo J. M. Riemens, Samantha Stora, Mathilde Roche, Markus Leber, Steffi Riedel-Heller, Michael Wagner, Martin Scherer, Aimé Ravel, Clotilde Mircher, Cecile Cieuta-Walti, Sophie Durand, Daniel L. A. van de Hove, Per Hoffmann, Alfredo Ramirez, Thomas Haaf, Nady El Hajj, André Mégarbané

**Affiliations:** 1 Institute of Human Genetics, Julius Maximilian University, Wuerzburg, Germany; 2 Division of Hematology and Oncology, Department of Internal Medicine II, University Hospital, Wuerzburg, Germany; 3 Department of Bioinformatics, Julius Maximilian University, Wuerzburg, Germany; 4 Department of Psychiatry & Neuropsychology, School for Mental Health and Neuroscience (MHeNs), Maastricht University, Maastricht, the Netherlands; 5 Department of Psychiatry and Psychotherapy, University Medical Center Göttingen, Göttingen, Germany; 6 Institut Jérôme Lejeune, CRB BioJeL, 37 rue des Volontaires, Paris, France; 7 Division of Neurogenetics and Molecular Psychiatry, Department of Psychiatry and Psychotherapy, University of Cologne, Medical Faculty, 50937 Cologne, Germany; 8 Department of Neurodegeneration and Geriatric Psychiatry, University of Bonn, 53127 Bonn, Germany; 9 Institute of Social Medicine, Occupational Health and Public Health, University of Leipzig, 04103 Leipzig, Germany; 10 German Center for Neurodegenerative Diseases (DZNE), 53127 Bonn, Germany; 11 Department of Primary Medical Care, University Medical Centre Hamburg-Eppendorf, 20246 Hamburg, Germany; 12 Department of Psychiatry, Psychosomatics and Psychotherapy, University of Wuerzburg, Wuerzburg, Germany; 13 Institute of Human Genetics, University of Bonn, 53127 Bonn, Germany; 14 Department of Genomics, Life & Brain Center, University of Bonn, 53127 Bonn, Germany; 15 Division of Medical Genetics, University Hospital and Department of Biomedicine, University of Basel, CH-4058 Basel, Switzerland; 16 College of Health and Life Sciences, Hamad Bin Khalifa University, Education City, Doha, Qatar

**Keywords:** Trisomy 21, DNA methylation, Intellectual disability, Down syndrome, Cognitive function, Alzheimer’s disease, Infinium Methylation EPIC arrays

## Abstract

**Abstract:**

**Background:**

Trisomy 21 (T21) is associated with intellectual disability that ranges from mild to profound with an average intellectual quotient of around 50. Furthermore, T21 patients have a high risk of developing Alzheimer’s disease (AD) early in life, characterized by the presence of senile plaques of amyloid protein and neurofibrillary tangles, leading to neuronal loss and cognitive decline. We postulate that epigenetic factors contribute to the observed variability in intellectual disability, as well as at the level of neurodegeneration seen in T21 individuals.

**Materials and Methods:**

A genome-wide DNA methylation study was performed using Illumina Infinium® MethylationEPIC BeadChips on whole blood DNA of 3 male T21 patients with low IQ, 8 T21 patients with high IQ (4 males and 4 females), and 21 age- and sex-matched control samples (12 males and 9 females) in order to determine whether DNA methylation alterations could help explain variation in cognitive impairment between individuals with T21. In view of the increased risk of developing AD in T21 individuals, we additionally investigated the T21-associated sites in published blood DNA methylation data from the AgeCoDe cohort (German study on Ageing, Cognition, and Dementia). AgeCoDe represents a prospective longitudinal study including non-demented individuals at baseline of which a part develops AD dementia at follow-up.

**Results:**

Two thousand seven hundred sixteen differentially methylated sites and regions discriminating T21 and healthy individuals were identified. In the T21 high and low IQ comparison, a single CpG located in the promoter of *PELI1* was differentially methylated after multiple testing adjustment. For the same contrast, 69 differentially methylated regions were identified. Performing a targeted association analysis for the significant T21-associated CpG sites in the AgeCoDe cohort, we found that 9 showed significant methylation differences related to AD dementia, including one in the *ADAM10* gene. This gene has previously been shown to play a role in the prevention of amyloid plaque formation in the brain.

**Conclusion:**

The differentially methylated regions may help understand the interaction between methylation alterations and cognitive function. In addition, *ADAM10* might be a valuable blood-based biomarker for at least the early detection of AD.

## Background

Trisomy 21 (T21) or Down syndrome is a chromosomal disorder resulting from the presence of all or part of an extra chromosome 21 that can be found free and homogeneous, in mosaicism, or as a translocation. It is a common birth defect occuring in one out of every 700-2000 newborns and is one of the most frequent forms of intellectual disability (ID) [1]. More than 100 characteristic features have been described in patients with T21 including physical, medical, and psychological features. ID is the most common feature present in 100% of the cases and ranges from mild to profound [2]. It is also well known that the brains of patients with T21 demonstrate high numbers of senile plaques and neuronal loss already at an early age of 40, a similar to what is seen in patients suffering from early onset AD, and commonly associated with an increased dosage of the amyloid precursor protein (*APP*) gene. Some patients with T21 will develop AD while some others will not [3].

Patients with T21 have different DNA methylation patterns compared to the general population [2]. This DNA methylation variation may partly explain phenotypic variations in T21 patients linked to premature aging, the concomitant development of AD, and negative neurodevelopmental effects, and as such, might represent a biomarker for T21-related neurodegeneration [3–12].

With this in mind, we postulated that DNA methylation variation might contribute to the level of ID and could help explain variation in cognitive impairment and dementia in T21. It is also still unclear why there are homogeneous T21 patients with severe ID despite the absence of any genetic or social causes [13]. We have previously identified downregulation of *HLA-DQA1* and *HLA-DRB1* in DS patients with severe ID after performing digital gene expression via SAGE on pooled RNA samples [1]. In non-DS patients, several studies have shown that genetic and epigenetic factors can explain to a large extent the variation in cognitive capacity [14–20].

Measuring DNA methylation in blood as a surrogate for potential changes in target/diseased tissues has its own limitations. Nevertheless, methylation measurements on easily accessible liquid biopsies can help identify biomarkers for diagnosis and risk stratification. Therefore, we performed a genome-wide DNA methylation study on T21 patients with high and low IQ as well as age- and gender-matched controls. We additionally compared the results to published data from the AgeCoDe cohort (German study on Ageing, Cognition, and Dementia) [21], a prospective longitudinal study focused on identifying risk factors of dementia, cognitive decline, and AD. Individuals who participated in the AgeCoDe study were healthy at time point 1 (T1). After clinical follow-up (4–5 years), individuals who developed AD at time point 2 (T2) were classified as converters whereas those who remained healthy were classified as non-converters. Since T21 patients may develop early-onset AD [12, 22–24], we performed a targeted association analysis using differentially methylated T21 CpG sites on blood methylation data from AD converters and non-converters at both time points. This approach was used to identify epigenetic markers directly related to AD dementia and not earlier AD pathology, as well as to find overlap in dysregulated genes that can be potentially associated with the development of AD-like pathology.

## Results

### Cell type composition

After measuring DNA methylation on whole blood DNA of the T21 cohort and control samples, we obtained methylation measurements for 850,000 CpG sites, where 33,661 sites were excluded from the analysis since they overlapped known SNPs or were located on sex chromosomes. First, we assessed the relative proportion of white blood counts on the basis of genome-wide methylation profiles using statistical methods [25]. We did not detect significant differences between T21 IQ− and T21 IQ+ cohorts, but we did observe differences between the T21 cohort and the controls (Additional file 1: Figure S1). Therefore, further analysis was adjusted for differential blood cell composition and gender in order to exclude possible effects on the observed methylation differences.

### DNA methylation changes in T21 patients

**Fig. 1:**
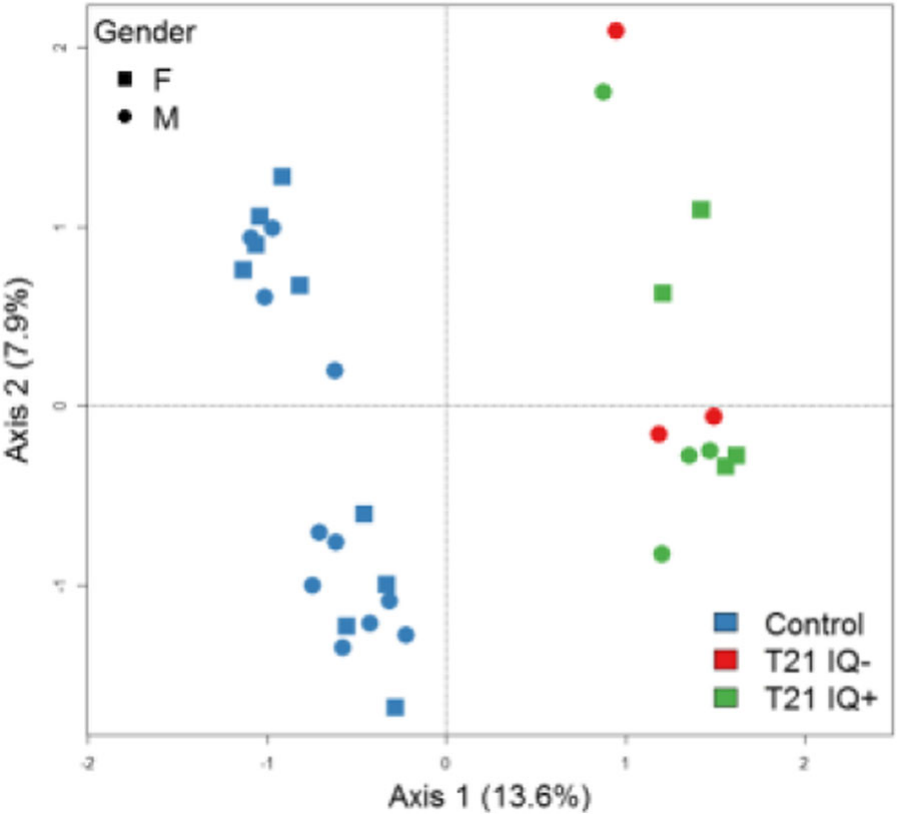
Correspondence analysis of the top 10,000 variable sites over all beta values. At each axis, the explained percentage of variation is denoted in parentheses

**Fig. 2:**
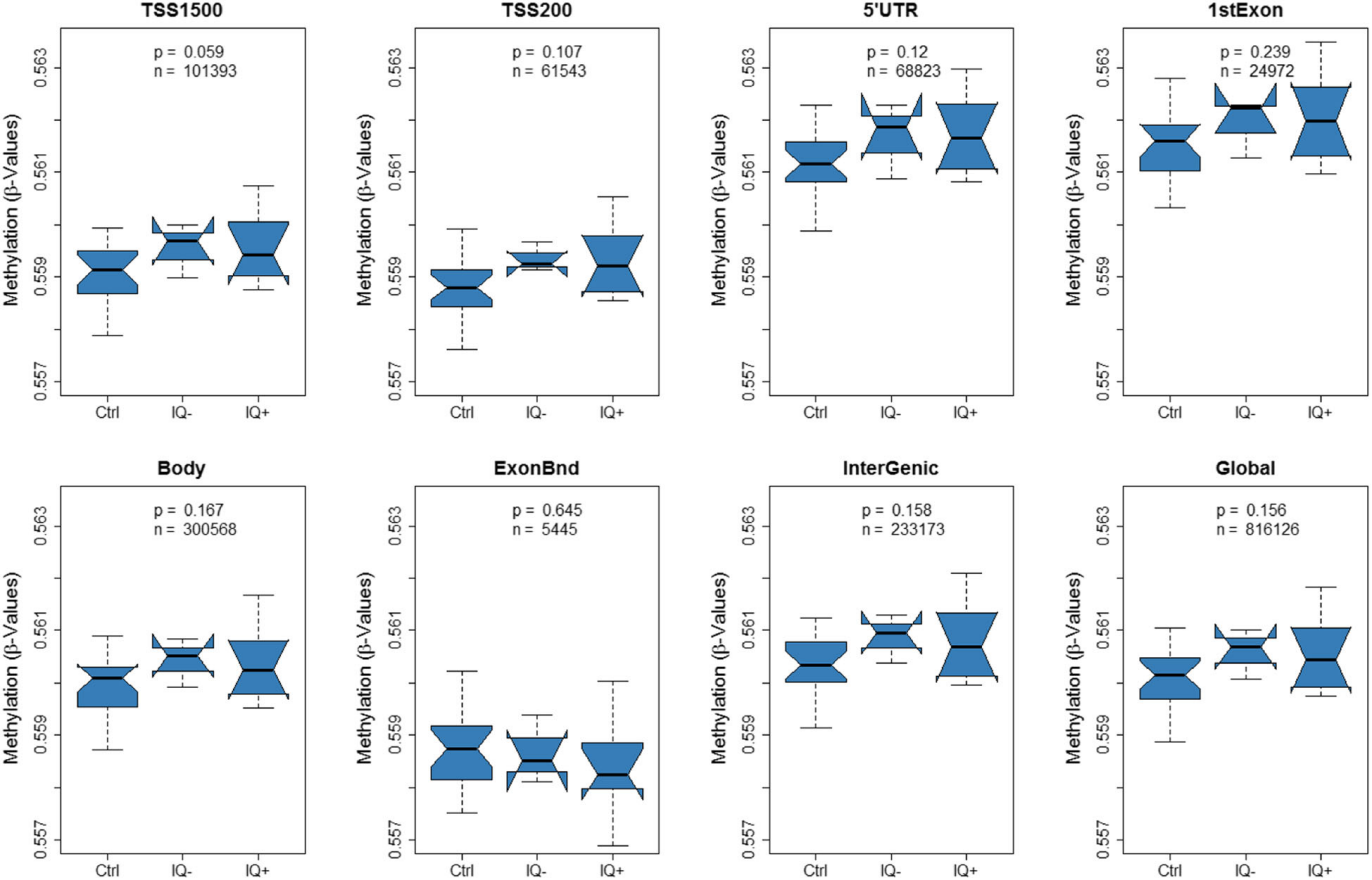
Global mean methylation analysis across various genomic features. In each boxplot, the total number of measured sites and the associated *p* value of the Kruskal-Wallis test comparing the mean methylation between the groups is reported

**Fig. 3:**
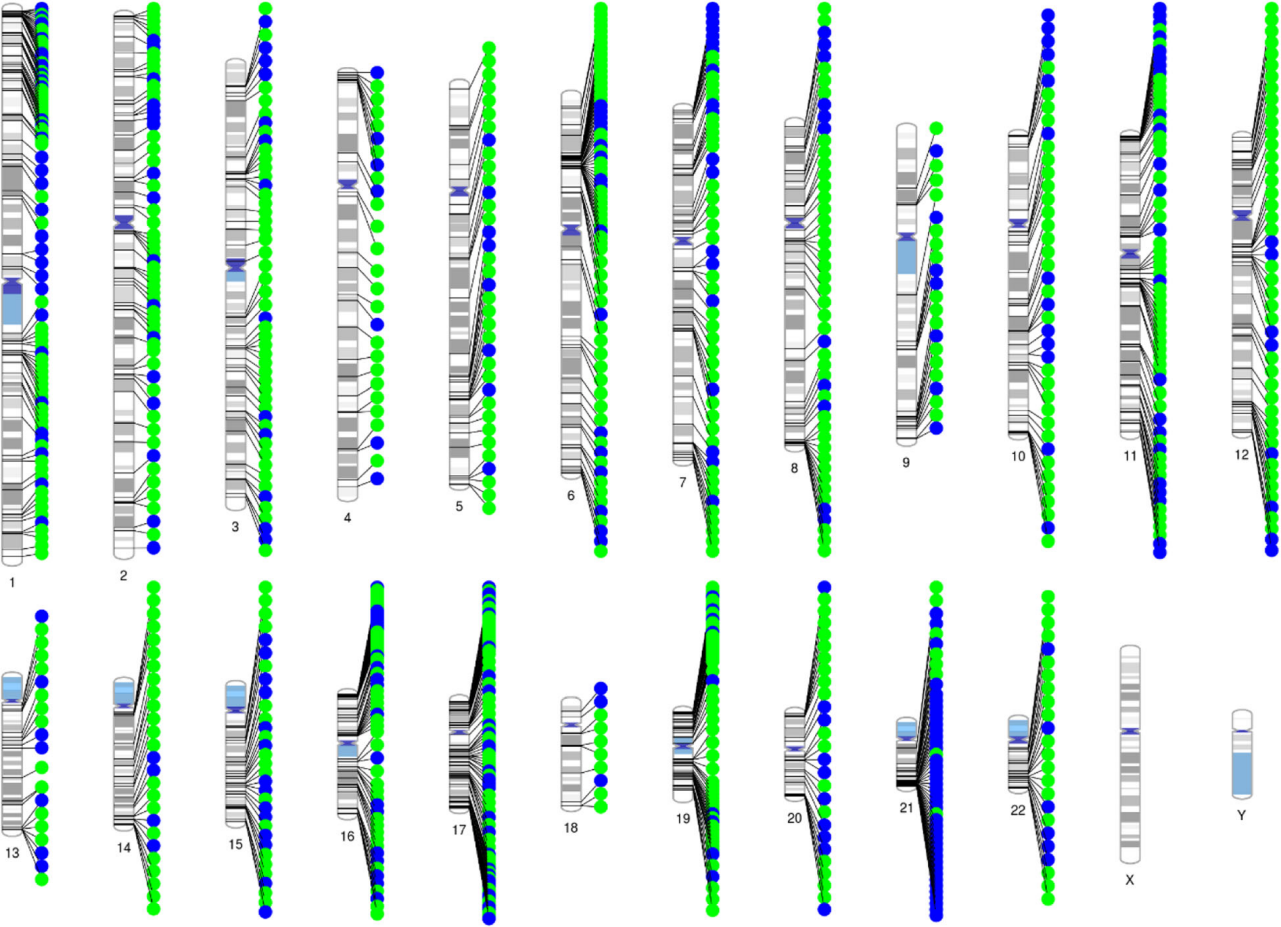
Chromosomal location of the top 1,000 differentially methylated clusters between Down’s syndrome patients and controls. Green dots indicate a hypermethylation in T21 samples whereas blue dots represent hypomethylated regions

A correspondence analysis revealed a clear effect of T21 on DNA methylation alterations (Fig. 1). In total, 35,609 (4.36%) of 816,126 analyzed CpGs exhibited significant methylation differences (FDR-adjusted *p* < 0.05) between T21 and controls after adjusting for confounding factors, i.e., blood cell composition and gender (Additional file 2: Table S1). We measured global methylation where we could observe a hypermethylation in T21 patients across the majority of genomic features apart from exon-boundaries (Fig. 2). Next, we performed a region-based analysis that revealed 2,716 DMRs between T21 and controls, out of which 80.7% were located within genes or promoter regions of genes (Additional file 2: Table S2). Several of these genes were previously reported to be differentially methylated in T21 patients. Significant DMRs were distributed across all chromosomes with an enrichment for chr21q22 (FDR-adjusted *p* value = 0.000027). Most DMRs on chromosome 21 were hypomethylated in T21 patients (Fig. 3). We then compared the 2,716 DMRs to a meta-analysis performed on T21 fetal brain, adult brain, placenta, epithelial tissue, and blood [26]. This comparison revealed that all genes apart of *ADAMTS10* and *LOC100130522* were significantly differentially methylated in our cohort (Additional file 2: Table S3).

### DNA methylation changes in low vs high IQ T21 patients

**Fig. 4:**
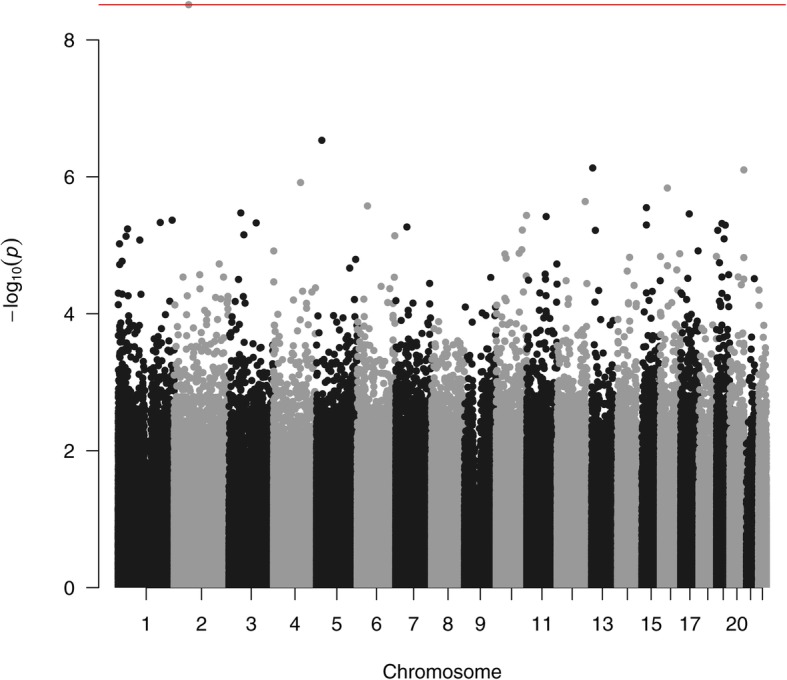
Manhattan plot of the differentially methylated CpG sites in the comparison of T21 patients with low and high IQ. The red line denotes the least significant site surviving multiple testing

We then compared the DNA methylation profile of T21 patients with IQ+ vs IQ−. Here, a CpG-based analysis revealed only one single differentially methylated CpG site, cg22352474, (Fig. 4) located in the promoter of *PELI1* on chromosome 2 (FDR-adjusted *p* value = 0.0025) (Table 1, Additional file 1: Figure S2). Subsequently, we performed a region-based analysis to detect CpG DMRs exhibiting differential methylation between the two groups. This revealed 69 significant DMRs showing differential methylation between T21 IQ+ and T21 IQ− (Additional file 2: Table S4). Those DMRs were located on all chromosomes apart from 14, 21, and 22. The top ranked DMR is hypomethylated in T21 IQ− and positioned in the phospholipase C beta 2 *(PLCB2)* gene on chromosome 15. In T21 IQ−, 43 DMRs (71.43%) were hypermethylated whereas 26 DMRs showed a hypomethylation. Next, we used Enrichr to determine whether differentially methylated genes are enriched for certain pathways. However, we detected no significantly enriched pathways after FDR-adjustment. None of the significant CpG sites in the region analysis for IQ− vs IQ+, nor the site in *PELI1*, was significantly altered in relation to AD.

**Table 1:** Top 10 differentially methylated CpGs within the T21 IQ-/T21 IQ+ contrast. Positive β differences indicate hypermethylation and negative β differences hypomethylation in the T21 IQ− group in comparison with the T21 IQ+ group

	Methylation difference	Average methylation	Adjusted *p* value	Genomic location	Gene	Region
cg22352474	0.07	0.06	0.002	chr2: 64371530	*PELI1*	5′UTR;1stExon
cg02307184	0.24	0.20	0.119	chr5: 23821294		
cg05755219	− 0.20	0.84	0.161	chr13: 24386298	*MIPEP*	Body
cg19166616	0.13	0.12	0.161	chr20: 62259876	*GMEB2*	TSS1500
cg13939234	0.11	0.08	0.198	chr4: 120375997		
cg26950080	0.06	0.75	0.198	chr16: 31162846	*PRSS36*	TSS1500
cg03159409	− 0.11	0.72	0.205	chr12: 124779144	*FAM101A*	5′UTR
cg14071023	− 0.26	0.78	0.205	chr6: 45901289	*CLIC5*	Body
cg19442201	− 0.12	0.65	0.205	chr15: 40572550		
cg10584271	− 0.24	0.59	0.205	chr3: 52814643	*ITIH1*	5′UTR;body

### Differentially methylated sites in the Alzheimer’s disease patients

**Fig. 5:**
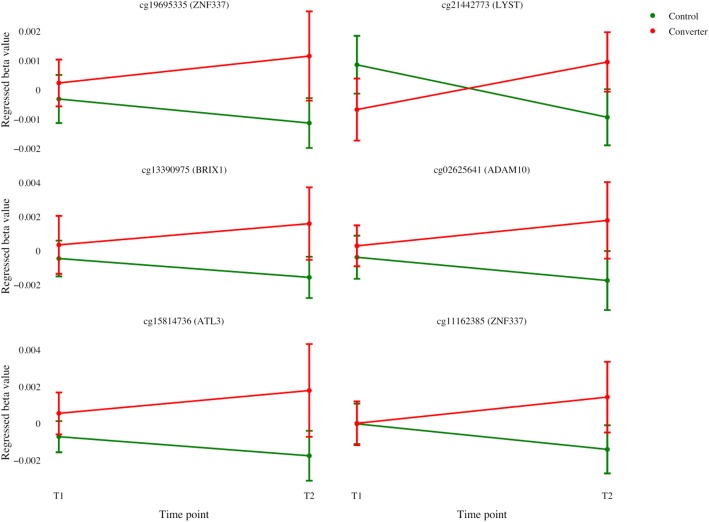
Mean regressed beta values and standard error of the mean (SEM) of Alzheimer’s disease (AD) dementia patients and controls before (T1) and after (T2) conversion to dementia, for the six differentially methylated CpG sites found in relation to both T21 and AD (and associated genes). The beta values were regressed using the same covariates as used for the association analysis

Next, we investigated the significant CpGs sites from the T21 patients and healthy controls comparison (Additional file 2: Table S1) in patients with AD dementia and controls, at time points before and after the development of dementia. Ten differentially methylated CpG sites (FDR-adjusted *p* < 0.05) between converters and non-converters at T2 were identified, that showed no methylation difference at T1 (Table 2), out of which six CpGs showed a similar methylation change in T21 and AD patients when compared to controls (Fig. 5). Pathway analysis using KEGG 2016, on the 5 genes associated with these 6 CpG sites, revealed enrichment for epithelial cell signaling in Helicobacter pylori (*p* value = 0.017) and AD (*p* value = 0.042). Furthermore, Panther 2016 analysis showed enrichment for AD-amyloid secretase pathway (*p* value = 0.014). The single gene involved in all those pathways was the A Disintegrin and metalloproteinase domain-containing protein 10 (ADAM10).

**Table 2:** List of differentially methylated CpGs with FDR-corrected *p* value < 0.05 in individuals who developed AD dementia in the AgeCoDe study. T2 is a comparison of AD patients (converters) vs controls (non-converters), whereas T1 is the same comparison before conversion to AD dementia. The 6 CpGs exhibiting a similar methylation change in DS and AD patients when compared to controls are highlighted in italic (LogFC: log2 fold change). Regulatory build is according to gene regulation data in Ensembl

	Gene	Regulatory build	logFC	Average	FDR	logFC	Average	FDR	LOGFC	FDR
			T2	T2	T2	T1	T1	T1	DS	DS
cg18431127	*EPB42*	TF binding site	*−* 0.115	0.452	0.003	0.004	0.468	0.980	0.049	0.016
*cg21442773*	*LYST*	Promoter	*0.018*	*0.064*	*0.003*	*− 0.003*	*0.064*	*0.939*	*0.091*	*0.010*
*cg19695335*	*ZNF337*	Promoter	*0.022*	*0.048*	*0.004*	*0.001*	*0.045*	*0.974*	*0.016*	*0.046*
*cg13390975*	*BRIX1*	Promoter	*0.030*	*0.052*	*0.005*	*0.001*	*0.048*	*0.979*	*0.107*	*0.001*
cg27004669	*MN1*	Open sea	*−* 0.113	0.439	0.005	0.017	0.448	0.897	0.048	0.018
*cg02625641*	*ADAM10*	Promoter	*0.034*	*0.081*	*0.009*	*0.001*	*0.077*	*0.978*	*0.032*	*0.013*
*cg1581473*6	*ATL3*	Promoter	*0.034*	*0.045*	*0.009*	*0.002*	*0.041*	*0.953*	*0.029*	*0.032*
*cg11162385*	*ZNF337*	Promoter	*0.027*	*0.071*	*0.012*	*0.000*	*0.068*	*0.996*	*0.044*	*0.014*
cg22599005	*CYP2W1*	CTCF	0.092	0.707	0.036	0.011	0.721	0.954	*−* 0.045	0.011

## Discussion

Even though the mechanisms of cognitive impairment in T21 are not completely understood, several reports point towards a complex interplay between genetic, epigenetic, and environmental factors contributing to the pathogenesis of ID in T21 patients [11, 27]. Here, we studied the methylation pattern of T21 patients with low and high IQ in addition to control individuals with a normal karyotype. The patients were free of any environmental factors or illness that might play a role in their cognitive function. Although several reports examined epigenetic differences in blood DNA of T21 patients, this is the first study to measure methylation differences using Infinium Methylation EPIC arrays that cover almost the double of CpGs compared to the 450K arrays. The EPIC beadchip platform contains > 90% of the CpG sites covered by the 450K array. A comparison of both platforms revealed high concordance and reproducibility of methylation measurements, which allows integration of EPIC and HM450K array data [28]. Our initial comparison of DNA methylation differences between T21 patients and controls yielded results largely identical to previously published studies [3, 29]. Here, we observed that 23 out of the 25 (92%) pan and multi-tissue T21 genes reported by Do et al. are differentially methylated in our study. One of the discordant genes, *ADAMTS10,* was not hypermethylated in adult T21 blood but rather in adult and fetal brain, as well as in epithelial tissue [26].

A cell type composition analysis using DNA methylation data revealed a significant increase in natural killer (NK) cells and decrease in B-lymphocytes, which is in agreement with previous studies performed using blood count testing [30–33]. A dysfunctional immune system is known to predispose T21 patients to various infections and autoimmune diseases. Infections are still considered one of the major causes of mortality in individuals suffering from T21 [34].

In T21 patients, we observed hypermethylation of the majority of differentially methylated CpG sites apart from chromosome 21, where an enrichment of hypomethylated CpGs was detected. We have previously identified a similar hypomethylation of chromosome 21 in fetal T21 brains [4]. This led us to apply different analysis approaches to determine whether the observed hypomethylation might be due to the applied normalization methods. Nevertheless, hypomethylation of chromosome 21 remained stable when normalizing via Dasen with and without BMIQ, Funnorm, and SWAN. Additionally, we observed increased expression in 6 of the 7 studied chromosome 21 genes. This unique methylation pattern has been similarly observed in other T21 studies on adult and fetal (cord) blood DNA [3, 35]. We still do not have a clear explanation of the biological basis of the observed dosage effect on the DNA methylation of chromosome 21. Several hypotheses have been proposed to explain the trans-acting epigenetic effects of an additional copy of chromosome 21. This includes dosage imbalance of chromosome 21 genes involved in epigenetic regulation including *DNMT3L*, which leads to increased methylation via stimulating the activity of *de novo* DNA methyltransferases (DNMTs) *DNMT3A* and *DNMT3B* [6, 11].

One aim of our study was to identify DNA methylation differences between IQ− and IQ+ T21 individuals. Since DNA methylation patterns are cell type-specific, it is important to identify constitutive DNA methylation changes that are shared across all tissues/cells. Cell type composition estimation for blood revealed similar cell type percentages which helped us delineate epigenetic group differences without cellular heterogeneity as a confounding factor.

Recently, Jones et al*.* reported that DNA methylation levels at certain loci are positively correlated with cognitive function in T21, as measured by the Dalton Brief Praxis test [10]. The authors used 450K methylation arrays to determine methylation changes in buccal swab cells of 10 T21 patients to identify CpG sites correlated with cognitive function. They identified 5 probes whose methylation measurements correlated with cognitive function, including 2 probes in the gene *TSC2*. We could not identify similar changes in our dataset which might be explained by the fact that we measured DNA methylation in a different tissue (blood and not buccal swab).

In our study, a single CpG site located in the promoter of *PELI1* survived multiple testing adjustments. The CpG site cg22352474 can have a possible role in the transcriptional regulation of *PELI1* as it is located in the promoter. The gene pellino E3 ubiquitin protein ligase 1 (*PELI1)* is a member of the Pellino family and is involved in mediating TLR3/TLR4 signaling [36]. *PELI* is abundantly expressed in microglia and has been shown to play a major role in regulating microglial activation in the central nervous system (CNS) [37]. Multiple reports have shown that microglial activation contributes to neuronal damage in neurodegenerative diseases [38, 39]. Evidence has also shown that neuro-inflammatory mechanisms may play a role in the pathophysiology of ID [40], and has been also associated with neurodegenerative disorders including multiple sclerosis and AD [41].

Since T21 patients may develop early-onset AD [12, 22–24], we investigated the significant CpG sites in T21 patients in subjects with AD dementia and controls, and found that 6 sites were hypermethylated in both T21 and AD patients versus healthy controls. One of these is located in the *ADAM10* promoter region, a gene that encodes a member of the ADAM (a disintegrin and metalloprotease) family, which has been identified as the constitutive α-secretase in the process of amyloid-β protein precursor (AβPP) cleavage, playing a role in reducing the generation of amyloid-β (Aβ) peptides [42]. No difference in methylation was found for *ADAM10* between the IQ+ and IQ− group, ruling out the role of this gene in cognition. It is important to mention that IQ in adults with T21 is not associated with risk or age at onset of AD [43]. Nevertheless, the results found in relation to AD dementia are in line with a recent report in AD brains, stating that the densities of cortical neurons expressing ADAM10 were significantly lower than in control subjects [44]. In confirmation to this, recent studies demonstrate a role for ADAM10 in the ectodomain shedding of low density lipoprotein receptor-related protein 1 (LRP1), a receptor responsible for the transport of Aβ in the brain and thus attenuating Aβ accumulation in the AD brain [45]. In addition, it was shown that variants located in the *ADAM10* locus increase the risk of late onset AD and that genetic variants affecting APP and Aβ processing are associated with early and late-onset AD [46].

Herein, the effect of genetic variants could be mediated by methylation changes which could explain the finding in AgeCoDe and could also reflect a potential biological mechanism which is mediated by the expression of substrates of ADAM10 such as APP which is increased in T21.

In the present study, T21 patients were on average 27 years old which is a relatively early age to have clinical AD features. It might be interesting to follow this cohort to check which of the patients will develop AD and confirm ADAM10 hypermethylation as a biomarker for the disease (at least in T21 patients). This aspect as well as the low number of IQ (−) T21 samples are limitations of the current study. Furthermore, one of the drawbacks of traditional bisulfite conversion techniques used in this study is their inability to differentiate 5-mC and 5-hmC. Future studies should implement the recently developed oxidative bisulfite conversion methods to distinguish different cytosine modifications.

## Conclusion

We have identified T21-related methylation patterns, as well as ID-related patterns in T21 patients with high and low IQ, and compared these with methylation profiles associated with AD dementia. We could identify a strong T21 effect while only a weak IQ effect was observed. Our analysis using arrays to measure DNA methylation differences in peripheral blood DNA identified several differentially methylated sites/regions which may help understand the interaction between methylation alterations, cognitive function, and AD. Follow-up studies should try to establish whether methylation at the identified loci would correlate with ID levels and AD in larger cohorts. If so, these loci may become valuable for the development of blood-based biomarkers for cognition and for the development of new drug targets.

## Materials and Methods

### Study subject and DNA preparation

We screened > 5500 clinical files collected at the Jérôme Lejeune Institute for DS patients with IQ > 70 (high IQ) and IQ < 20 (low IQ). To eliminate factors that might influence the cognitive function, we did not include patients that had thyroid abnormalities, heart problems, cancer, hearing problems, vision impairment, neurological problems (epilepsy, seizures, west syndromes, etc.), autism, diabetes, social problems, changes indicating early dementia, sleep-disordered breathing, or were under any medication. Furthermore, no serious events such as child abuse, frequent hospitalization, and death of one of the parents or siblings were reported in the families of the patients. In total, we identified 8 DS patients with IQ > 70 and 3 DS patients with IQ < 20 where whole blood DNA was collected at the Jérôme Lejeune Institute. All selected patients had a homogeneous trisomy 21 in all cells with no mosaicism or translocation.

Patients with an age between 19 and 34 years (mean 27.9 years) were subdivided in two groups with a lower IQ (IQ < 20 or IQ−; three males and no female) and a higher IQ (IQ > 70 or IQ+; four males and four females), respectively. IQ measurement was performed with the Columbia test. Only patients without known comorbidities, medications and with negative family history were selected. Whole blood samples of aged-matched healthy individuals (12 males and 9 females) were collected at the Institute of Human Genetics of the University of Wuerzburg. The age of the control group ranged from 21 to 34 years (mean: 27.5 years). Genomic DNA was isolated immediately after collecting the blood by using the FlexiGene DNA Kit (Qiagen, Hilden, Germany) according to the provided manual. Amount and quality of the DNA were measured with the Qubit dsDNA BR Assay Kit (Thermo Fisher Scientific, Waltham, USA) and the NanoDrop 2000c spectrophotometer (Thermo Fisher Scientific). All participants were of middle European descent.

The AgeCoDe cohort enrolled and longitudinally followed 3,327 non-demented individuals at baseline. This study was initiated to investigate methods and biomarkers for early identification of dementia and mild cognitive impairment. Randomly selected subjects were recruited in six German cities, and cognition level was evaluated for up to 11 years after enrollment. A cognition test was performed every 18 months until the 7th visit where cognition was assessed in intervals of 10 months. From this cohort, we identified 42 individuals > 75 years old, healthy at baseline, and diagnosed with AD dementia (converters) after ~ 4.5 years. Similarly, 42 age, gender, and APOE genotype-matched individuals with no signs of dementia at baseline or follow-up were selected as controls (non-converters). Whole blood DNA was collected at both baseline and follow-up from both converters and non-converters. Dementia was diagnosed using the Structured Interview for Diagnosis of Dementia of Alzheimer Type, Multi-infarct Dementia, and Dementia of Other Etiology according to the DSM-IV criteria. The Blessed Dementia Rating subscales and the Global Deterioration Scale [47] (> = 4) were used to assess dementia presence in individuals who were not interviewed by a health care practitioner. Alzheimer’s disease was diagnosed according to the guidelines of the Stroke and the Alzheimer’s Disease and Related Disorders Association as well as the National Institute of Neurological and Communicative Disorders [48] only if sufficient clinical evidence was present. A consensus of both the interviewer and an experienced geriatrician or geriatric psychiatrist determined the final diagnoses for AD in all converters [49, 50]. At follow-up, the study size was 42 individuals for both groups (32 females plus 10 males as non-converters and 29 females plus 13 males as converters).

### Methylation array

For the T21 patients and the control group, sodium bisulfite conversion was performed using the EZ DNA Methylation™ Kit (Zymo Research, Irvine, CA, USA) according to the manufacturer’s instructions (500 ng DNA each sample). Samples of the AgeCoDe study were converted with the Qiagen EpiTect 96 Bisulfite Kit (Qiagen, Hilden, Germany) [21].

The AgeCoDe study was conducted with Infinium HumanMethylation450K arrays (Illumina, San Diego, CA), while the T21 IQ comparison was performed with Infinium® MethylationEPIC BeadChips. The latter allows quantification of more than 850,000 CpG sites across the genome including promoters, CpG islands, gene bodies, and enhancer regions. After whole-genome amplification and enzymatic fragmentation, the samples were hybridized to 4 BeadChips and scanning was conducted with the Illumina iScan ((NCBI GEO accession no GSE140344). To avoid batch effects, all BeadChips were processed simultaneously and the samples were gender- and affection-matched. Idat files were exported and analyzed with the R software package (version 3.2.2) and the BioConductor platform (version 3.2). Data preprocessing was done using the minfi [51] package. Cross-hybridizing probes and probes overlapping known SNPs and those on the sex chromosomes were removed. In total, 816,126 probes met all quality criteria and were used for subsequent analyses. Intensity values were normalized using the quantile normalization procedure as implemented in the minfi package. Based on the methylation profiles of cell-type specific CpGs, blood cell composition was estimated [25]. Differential methylation analysis has been performed using the moderated *T*-test model as implemented in the *limma* package [52] based on *β* values adjusting for cell composition and gender. Multiple testing corrections were performed for all *p* values with the Benjamini-Hochberg method. Cell composition between the T21 group and the controls were compared by Wilcoxon-Mann-Whitney test. Correspondence analysis was performed as implemented in the vegan package. To derive differentially methylated regions (DMRs) from probewise *p* values, we used the approach implemented in the comb-*p* package [53]. In general, this approach comprises three steps: first, a Stouffer-Liptak-Kechris (SLK)-corrected *p* value for each probe is calculated based on the autocorrelation on neighboring *p* values. In a second step, regions enriched with SLK-corrected *p* values were identified by a peak-finding algorithm. Finally, the significance of each identified region is then determined by applying a Stouffer-Liptak correction to the original *p* values of all probes in the region. To correct for multiple testing, a Šidák correction, based on the number of possible regions of the same size, is applied to all identified regions. A region is extended if another *p* value within a genomic distance of 1000 nucleotides is found (dist = 1000). Sites with a *p* value < 0.05 (seed = 0.05) were considered as a starting point for a potential region.

For the AgeCoDe samples, computational and statistical analyses were performed in a similar manner as described earlier. The “pfilter” function of the *wateRmelon* package (version 1.18.0) was used for probe filtering (1351 probes were removed). The remaining probe data was normalized using the *dasen* method, as implemented in the *wateRmelon* package. The gender of the samples was predicted based on X chromosome methylation using the *DNAmArray* package (version 0.0.2), compared with the assumed gender, and mismatches were excluded (*N* = 2). After data processing, 97 blood samples remained, with 402,561 remaining probes in the blood datasets. The case-control analysis of the blood follow-up data included 84 samples, including the 42 converters that had already converted to AD at the 4.5-year follow-up and excluding those that had converted later. Next, a genetic fingerprinting test based on 65 SNP probes located on the HM 450K chip [54] was applied to confirm that the matching T1 and T2 DNA samples were from the same individual. This fingerprinting test identified 2 donors with mismatching samples, which were excluded from further analysis. A surrogate variable (SV) analysis was performed with the *sva* package (version 3.22.0) [55] with AD conversion as predictor, age and gender as covariates, and beta values as outcome. To adjust for unobserved confounders, the first SVs of this analysis were added to the model and replaced with the HMK chip IDs. A linear regression analysis was done to test the association between AD conversion and beta values. Test statistics were adjusted for bias and inflation with the *bacon* package (version 1.2.0) [56]. FDR correction for multiple testing was performed, and individual probes were annotated using Illumina UCSC annotation. Results from the statistically significantly T21-associated candidate probes were then extracted from the AD blood analysis before and after conversion, and *p* values were readjusted for this subset. To specifically identify dementia-related probes, only probes showing a difference in methylation after conversion, but not before, were selected. Gene set enrichment analysis including KEGG and Panther analysis was performed via the Enrichr tool (http://amp.pharm.mssm.edu/Enrichr/) [57, 58].

## Supplementary information

**Additional file 1: Figure S1.** Boxplots of estimated blood cell composition based on methylation array profiles of cell-type-specific CpGs. Median is represented by a horizontal line. The top of the box indicates the 75th percentile, the bottom the 25th percentile. Black dots represent outliers. The Y-axis shows the percentage of a given cell type. On the X-axis C indicates controls and T trisomy 21 patients. The table indicates the (FDR-adjusted) p-values of a Wilcoxon-Mann–Whitney test comparing the means between controls and T21 patients. **Figure S2.** cg22352474 is located within a CpG island in the promoter region of the *PELI1* gene. The figure was adapted according to ENSG00000197329 (ENSMBL release GRCh38.p12).

**Additional file 2: Table S1.** CpGs DS vs Ctrl. **Table S2.** DMR DS vs Ctrl. **Table S3.** This table is adapted from a gene list presented in Fig. 3c by Do et al. (2017). **Table S4.** DMR IQ- vs IQ+.

## Data Availability

The EPIC array data are uploaded to NCBI GEO and are available under accession number GSE140344 (or even before if necessary). For the AgeCoDe data, it is not publicly available as other paper(s) are submitted and not yet accepted, but available from the corresponding authors on reasonable request. Ethics approval and consent to participate The study protocols were approved by the ethical committee of the Jérôme Lejeune Institute and its scientific council. Written informed consent was obtained from the T21 patients or from the legal representatives when applicable by the medical staff of the Jérôme Lejeune Institute. The latter confirms that his research center possesses the authorizations for biobanking activities (AC-2015-2579), and for human samples exportation (IE-2015-814). The AgeCoDe study protocol was approved by the local ethics committees at the University of Bonn (Bonn, Germany), the University of Hamburg (Hamburg, Germany), the University of Duesseldorf (Duesseldorf, Germany), the University of Heidelberg/Mannheim (Mannheim, Germany), the University of Leipzig (Leipzig, Germany), and the Technical University of Munich (Munich, Germany). Written informed consent from all participating individuals, parents, or guardians on behalf of the participants unable to provide consent was obtained, and the study was performed following the guidelines of the Declaration of Helsinki.

## Abbreviations

T21: Trisomy 21
IQ: Intellectual quotient
ID: Intellectual disability
AD: Alzheimer’s disease
T1/2: Time point 1/2
