# Supplementary material for: Methylomic profiling in trisomy 21 identifies cognition- and Alzheimer’s disease-related dysregulation
Source: Clin Epigenetics. 2019 Dec 16;11:195. doi: 10.1186/s13148-019-0787-x (PMC6916110; doi:10.1186/s13148-019-0787-x)
Supplement: Supplementary file 1 — Additional file 1: Figure S1. Boxplots of estimated blood cell composition based on methylation array profiles of cell-type-specific CpGs. Median is represented by a horizontal line. The top of the box indicates the 75th percentile, the bottom the 25th percentile. Black dots represent outliers. The Y-axis shows the percentage of a given cell type. On the X-axis C indicates controls and T trisomy 21 patients. The table indicates the (FDR-adjusted) p-values of a Wilcoxon-Mann–Whitney test comparing the means between controls and T21 patients. Figure S2. cg22352474 is located within a CpG island in the promoter region of the PELI1 gene. The figure was adapted according to ENSG00000197329 (ENSMBL release GRCh38.p12). [file 13148_2019_787_MOESM1_ESM.docx]

**Supplementary Figures:**


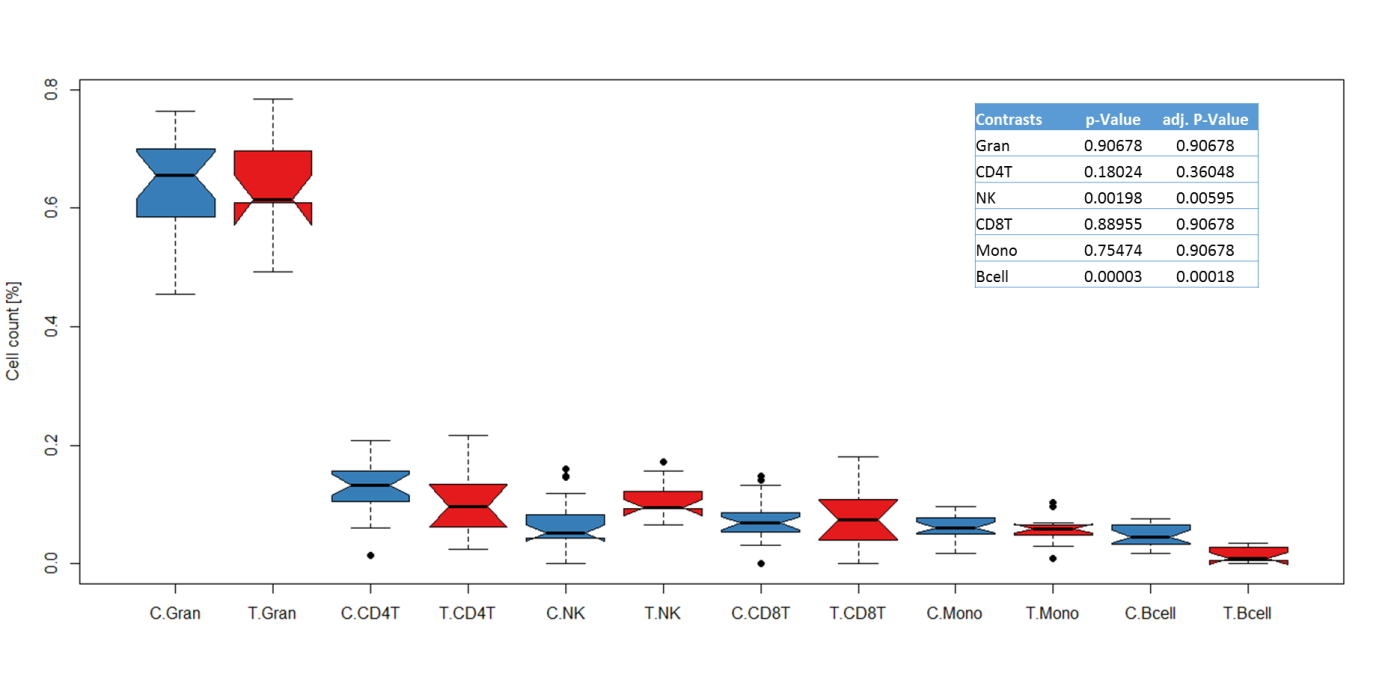


**Additional file 1: Figure S1.** Boxplots of estimated blood cell composition based on methylation array profiles of cell-type-specific CpGs. Median is represented by a horizontal line. The top of the box indicates the 75th percentile, the bottom the 25th percentile. Black dots represent outliers. The Y-axis shows the percentage of a given cell type. On the X-axis C indicates controls and T trisomy 21 patients. The table indicates the (FDR-adjusted) p-values of a Wilcoxon-Mann–Whitney test comparing the means between controls and T21 patients.


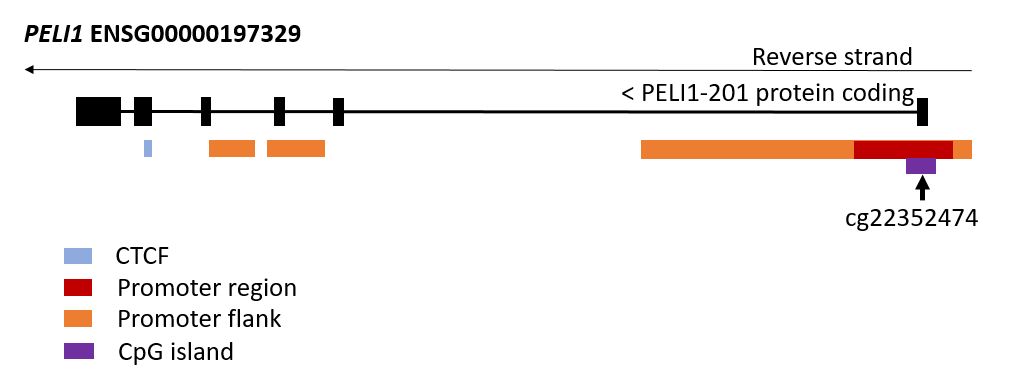


**Additional file 1: Figure S2:** cg22352474 is located within a CpG island in the promoter region of the *PELI1* gene. The figure was adapted according to ENSG00000197329 (ENSMBL release GRCh38.p12).
